# Water‐Soluble Aggregation‐Induced Emission Luminogens with Near‐Infrared Emission for Advanced Phototheranostics

**DOI:** 10.1002/smsc.202300052

**Published:** 2023-07-21

**Authors:** Hanchen Shen, Changhuo Xu, Ruquan Ye, Tzu-Ming Liu, Jianyu Zhang, Ryan T. K. Kwok, Jacky W. Y. Lam, Zhihong Guo, Jianwei Sun, Ben Zhong Tang

**Affiliations:** ^1^ Department of Chemistry Hong Kong Branch of Chinese National Engineering Research Center for Tissue Restoration and Reconstruction Division of Life Science State Key Laboratory of Molecular Neuroscience The Hong Kong University of Science and Technology Clear Water Bay Kowloon Hong Kong 999077 China; ^2^ MOE Frontiers Science Center for Precision Oncology Faculty of Health Sciences University of Macau Macao 999078 China; ^3^ Department of Chemistry State Key Laboratory of Marine Pollution City University of Hong Kong Hong Kong 999077 China; ^4^ School of Science and Engineering Shenzhen Institute of Aggregate Science and Technology The Chinese University of Hong Kong Shenzhen Guangdong 518172 China; ^5^ Center for Aggregation-Induced Emission South China University of Technology Guangzhou 510640 China

**Keywords:** aggregation-induced emission, bioprobes, fluorescence, good water solubility, near-infrared emission, phototheranostics

## Abstract

The development of water‐soluble aggregation‐induced emission luminogens (AIEgens) emitting in the near‐infrared (NIR) window holds promise for efficient biomedical applications. Nevertheless, synthesizing water‐soluble counterparts of NIR AIEgens presents difficulties due to their intrinsic hydrophobic properties. To address this issue, researchers have developed various molecular design strategies to improve the water solubility of NIR AIEgens. The integration of hydrophilic groups and targeting moieties is a crucial aspect of achieving precise phototheranostics. Here, diverse approaches to attain water‐soluble NIR AIEgens for biomedical applications are presented, and three commonly used strategies that involve decorating NIR AIEgens with positively or negatively charged groups, hydrophilic chains, and bioactive moieties are elaborated. These rational design strategies are believed to provide solutions for enhancing the water solubility and biological performance of NIR AIEgens in a single action. The remaining challenges and opportunities in this field are also discussed. The aim is to provide new insights into the design of water‐soluble NIR AIEgens and inspire more researchers to make significant contributions to this promising research area.

## Graphical abstract

Insights are offered into the design strategies for water‐soluble near‐infrared (NIR) aggregation‐induced emission luminogens (AIEgens) and their biomedical applications. The remaining challenges and future directions for the development of water‐soluble NIR AIEgens are also explored. The aim of this work is to inspire more researchers to make efforts in this promising area.

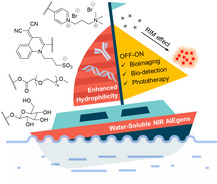

## 1 Introduction

The exploration of light–matter interactions has fueled the development of novel photoactive materials,^[^ ^1^, ^2^, ^3^, ^4^, ^5^, ^6^, ^7^ ^]^ which are continuously revolutionizing the world with their emerging applications, such as imaging, sensing, and communication. Among them, fluorescence (FL) technology has emerged as a powerful tool for biomedical applications because it allows researchers to visualize biological processes with high sensitivity and specificity. FL imaging has gained popularity in recent years due to its distinct benefits, including high sensitivity, minimal invasiveness, ease of use, low cost, and satisfactory biocompatibility among all the frequently employed imaging modalities.^[^ ^8^, ^9^ ^]^ Organic fluorescent dyes are categorized as an indispensable kind of FL imaging agent in terms of their great diversity and superior performance. However, the luminescent properties of many organic dyes differ significantly in the single‐molecule and aggregate states. This feature can limit their use in physiological environments where dye aggregation is inevitable.^[^ ^10^, ^11^ ^]^ A representative case is the notorious phenomenon of aggregation‐caused quenching, where the aggregation of fluorescent dyes leads to weak or loss of FL signals in the physiological environment to impede their applications.^[^ ^11^ ^]^ In contrast, aggregation‐induced emission (AIE) is a phenomenon where luminogens emit faintly in the solution state, but exhibit enhanced FL in the aggregate state.^[^ ^12^, ^13^ ^]^ Researchers have explored various mechanisms to explain the AIE phenomenon. These mechanisms can be generalized to the restriction of intramolecular motion (RIM), which blocks the non‐radiative decay pathway of AIE luminogens (AIEgens) in the aggregate state and allows excitons to decay radiatively.^[^ ^14^, ^15^, ^16^ ^]^ AIEgens show unique advantages, such as high brightness, large Stokes shift, good biocompatibility, and satisfactory photostability.^[^ ^17^, ^18^, ^19^, ^20^, ^21^, ^22^ ^]^ Moreover, some AIEgens can also act as efficient therapeutic agents by generating reactive oxygen species (ROS) for photodynamic therapy (PDT) or excessive heat for photothermal therapy.^[^ ^23^, ^24^, ^25^, ^26^, ^27^, ^28^ ^]^ In the aggregate state, non‐radiative decay of AIEgen is typically suppressed through the restriction of molecular motion.^[^ ^29^ ^]^ The suppressed non‐radiative decay facilitates efficient intersystem crossing and enables the generation of ROS, which can effectively kill cancer cells or bacteria. Conversely, by promoting the molecular motions of AIEgens in the aggregate state, they can exhibit strong photothermal conversion capabilities.^[^ ^24^ ^]^ Researchers can skillfully manipulate molecular motions to design potent photothermal agents with high photothermal conversion efficiency or multifunctional phototherapeutic agents with balanced photothermal effects. As a result, AIEgens have emerged as a promising therapeutic option for disease treatment.

In contrast, water‐soluble fluorescent dyes are desirable for effective biomedical applications as water solubility can impact their biological compatibility, sensitivity, and targeting specificity.^[^ ^30^ ^]^ Hydrophilization of AIEgens is thus vital for their practical bioapplications. So far, many strategies have been developed to disperse or solvate AIE materials in the aqueous environment. A popular way is to use organic and inorganic matrices to encapsulate AIEgens to fabricate AIE nanoparticles. One of the most commonly used organic nanoparticle matrices is 1,2‐distearoyl‐sn‐glycero‐3‐phosphoethanolamine–poly(ethylene glycol) (DSPE–PEG), which is preferred for its biosafety, stability, and modifiability.^[^ ^31^ ^]^ Water‐soluble polystyrene nanoparticles are widely used as dependable carriers of AIEgens for biodetection.^[^ ^32^ ^]^ Recently, other rigid organic matrices have been developed for the payload of AIEgens to achieve bright emission, such as calix[5] arene and corannulene‐decorated PEG.^[^ ^33^ ^]^ The utilization of inorganic materials to disperse AIEgens is also well developed. Silicon dioxide (SiO_2_) nanoparticles are broadly utilized as carriers for organic fluorophores in various biomedical applications because they offer numerous benefits, such as enhanced stability and biocompatibility.^[^ ^34^ ^]^ Interestingly, the SiO_2_ network can provide a rigid framework that can suppress the molecular movements of organic dyes to increase the brightness of AIEgens.^[^ ^35^ ^]^ Moreover, other emerging inorganic nonmetallic materials, such as 2D materials, are also developed as promising matrices. For example, graphene oxide loaded with AIEgens has been extensively employed for biodetection and phototherapeutic applications.^[^ ^36^, ^37^, ^38^, ^39^ ^]^ Recent research has also explored the combination of black phosphorus with AIE materials for advancing phototheranostic techniques by utilizing the unique advantages of 2D materials, such as large surface area, good aqueous dispersity, and high photothermal conversion efficiency.^[^ ^40^ ^]^

In addition to using organic or inorganic matrices for physical encapsulation, the rational design of water‐soluble AIEgen‐conjugated systems attracts even more attention because of their clear pharmacokinetics. A macromolecule‐based strategy to prepare water‐soluble single‐component AIE materials is to polymerize AIE‐active monomers to serve as the hydrophobic domain of amphiphilic block polymers or graft multiple AIE‐active moieties to natural or synthetic hydrophilic polymers.^[^ ^41^ ^]^ This strategy is promising because the versatility of polymer chemistry allows the robust incorporation of AIEgens to create functional polymeric architectures. Another common strategy is to decorate one AIEgen with hydrophilic moieties so that the resulting small‐molecule systems are structurally well defined. In most cases, the supreme water solubility of these systems allows adequate molecular motions in the physiological environment to quench their emission. Particular biomarkers can specifically reactivate their FL and PDT effects, thus achieving precise phototheranostics. However, if the hydrophobicity of the AIEgen prevails over the hydrophilicity of the polar moiety, a self‐assembly process would occur to restrict the molecular motion and lead to bright emission.^[^ ^42^ ^]^ Such materials are also important because of their small‐molecule‐like targeting effect and pharmacokinetics.

Small‐molecule water‐soluble AIEgens with well‐defined structures have a high potential for clinical applications due to several key advantages over other imaging agents, including small size, ease of use, clear pharmacokinetics, and chemical tunability.^[^ ^43^ ^]^ Researchers have made great efforts to develop water‐soluble AIEgens for biochemical analysis.^[^ ^44^, ^45^, ^46^, ^47^, ^48^, ^49^, ^50^, ^51^ ^]^ However, many of them emit in the visible‐light region with short‐emission wavelengths. Theoretically, FL signal in the near‐infrared (NIR) window (NIR‐I: 620–900 nm; NIR‐II: 1000–1700 nm) can penetrate deeper into biological tissues than visible light because of the reduced interface reflection, autofluorescence, and photon scattering.^[^ ^18^, ^52^, ^53^ ^]^ Developing water‐soluble NIR AIEgens is still challenging because current luminescent materials with redshifted emission typically show a large hydrophobic *π*‐conjugated molecular backbone and thereby their water solubility is much sacrificed.^[^ ^54^ ^]^ Fortunately, this dilemma can be overcome by rational molecular design to make water‐soluble NIR AIE‐active dyes synthetically accessible. In this perspective, we summarized the design strategy and recent progress of water‐soluble NIR AIEgens and their applications in phototheranostics. It should be noted that there are some discrepancies in the definition of NIR wavelength range among literature sources. This paper will exclusively focus on water‐soluble AIEgens with emission peaks located around or longer than 600 nm.

## 2 Design Strategy of Water‐Soluble NIR AIEgens

Most water‐soluble AIEgens exhibit a unique FL turn‐on property upon physical or chemical interactions with biological analytes. When they are molecularly dissolved in water, their vivid molecular motion will promote non‐radiative decay to confine them in the FL‐off state. Once interacted with the analytes, the strong RIM effect between AIEgens or between AIEgens and biomolecules will block the non‐radiative decay pathway to lead to strong FL. In addition to precise detection, it is important to note that such a turn‐on property of AIEgens has the potential to achieve precise PDT if they can produce ROS upon light irradiation. While traditional PDT treatment requires patients to remain in a dark room to avoid side effects induced by photosensitizers, AIE photosensitizers could minimize these side effects by only being activated at disease sites.^[^ ^55^ ^]^ This is because only photosensitizers activated by biomarkers at these sites would generate ROS to minimize potential adverse effects. Moreover, it has been reported that ROS generated at specific organelles can lead to more potent cytotoxicity, making AIE photosensitizers a promising option for both minimizing side effects and improving the effectiveness of PDT.^[^ ^56^ ^]^ Thus, stimuli‐responsive AIE photosensitizers have the potential to revolutionize the way of intraoperative PDT and improve patient outcomes.

**Figure 1:**
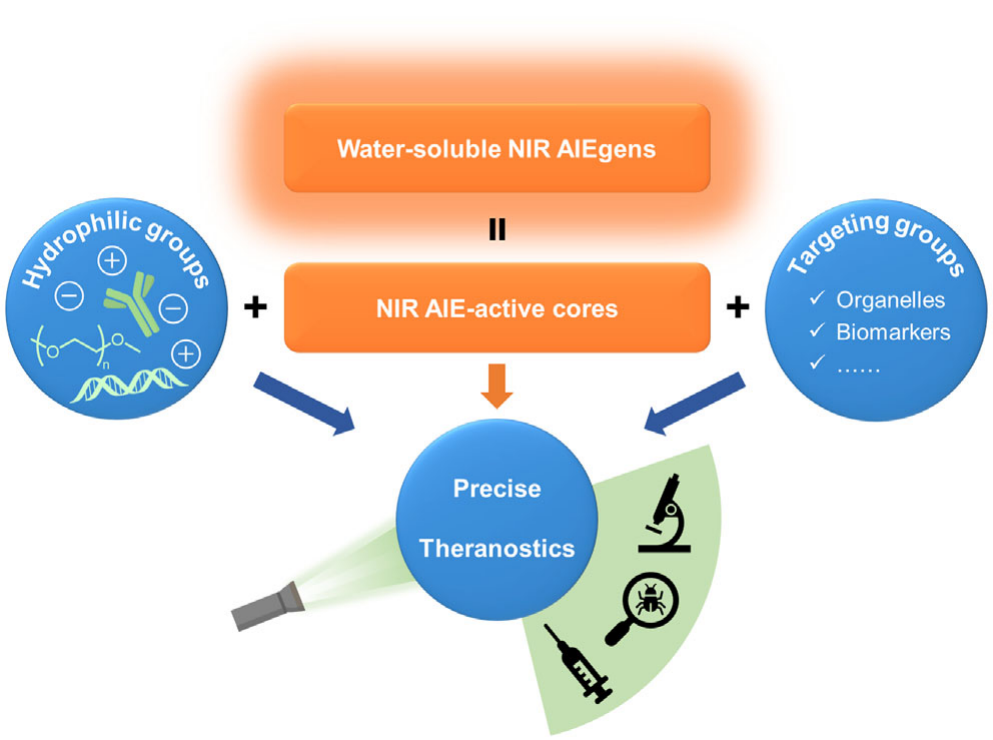
Schematic illustration of the molecular design of water‐soluble near‐infrared (NIR) aggregation‐induced emission luminogens (AIEgens).

Achieving precise phototheranostics requires both high water solubility and specific targeting ability to organelles or biomarkers (**Figure** **1**). Meanwhile, achieving this goal demands NIR emission from AIEgens.^[^ ^57^, ^58^, ^59^ ^]^ Two strategies are accessible to shift the emission wavelength of organic fluorophores into the NIR window.^[^ ^10^ ^]^ The first strategy is to extend the conjugation length. However, it is not practically available due to tedious synthesis, unsatisfactory processability, and instability. Another strategy is to develop donor–acceptor (D–A)‐structured AIEgens. The D–A strategy can effectively reduce the bandgap through relatively simple synthesis. Due to the typical twisted structures of AIEgens, D–A structures are commonly used to prepare NIR AIEgens. Frequently used electron donors to construct AIE‐active structures include triphenylamine, tetraphenylethylene, and dimethylamino groups, and electron acceptors include electron‐neutral benzothiadiazole, benzobisthiadiazole, 6,7‐diphenyl‐[1,2,5]thiadiazolo[3,4‐g]quinoxaline, and malononitrile derivatives. Positively charged pyridinium salts, acridinium salts, and indole derivatives are also adopted as electron acceptors. Moreover, a *π*‐bridge, such as alkylated thiophene, is introduced to modulate the chemical conjugation or spatial configuration to obtain the desired emission wavelength and FL quantum yield.^[^ ^17^ ^]^ It should be noted that although the D–A strategy does not require extended conjugation length, the donor and acceptor units are usually conjugated structures to make most NIR AIEgens highly hydrophobic. Therefore, moieties with good hydrophilicity are required to integrate with the hydrophobic core to endow the whole molecule with water solubility.

Many strategies have been proposed to improve the hydrophilicity of NIR AIEgens, including: 1) introduction of charged groups into AIEgens, such as pyridinium salts, quaternary ammonium salts, or sulfonate ions; 2) the use of hydrophilic chains, such as PEG and oligoethylene glycol (OEG), which are easy to synthesize with high chemical stability and electrically neutral character; and 3) incorporation of hydrophilic biomolecules such as carbohydrates, antibodies, and nucleic acids. In contrast, there are many options for targeting moieties for specific activation by biological analytes. Certain hydrophilic groups have intrinsic targeting abilities. For example, pyridinium or quaternary ammonium salts can target organelles like mitochondria or cell membranes.^[^ ^60^, ^61^ ^]^ Biomolecules like antibodies can interact with specific receptors to enable targeted delivery to tumor cells. In some cases, water‐soluble NIR AIEgens have separated hydrophilic and recognition moieties. In the following parts, we will categorize water‐soluble NIR AIEgens based on different hydrophilic groups and discuss their molecular design and biomedical applications.

## 3 Charged AIEgens

The most commonly used method to prepare water‐soluble NIR AIEgens is the introduction of positively and negatively charged groups. The rationale behind this approach is that the charged groups can be hydrated by polar water molecules, thereby increasing the water solubility of the AIEgens. Additionally, the molecular engineering of charged groups on AIEgens can also influence their bio‐distribution and targeting effect by altering their interactions with biomolecules. In this section, we will explore using positively and negatively charged AIEgens for biological applications.

### 3.1 Positively Charged AIEgens

**Figure 2:**
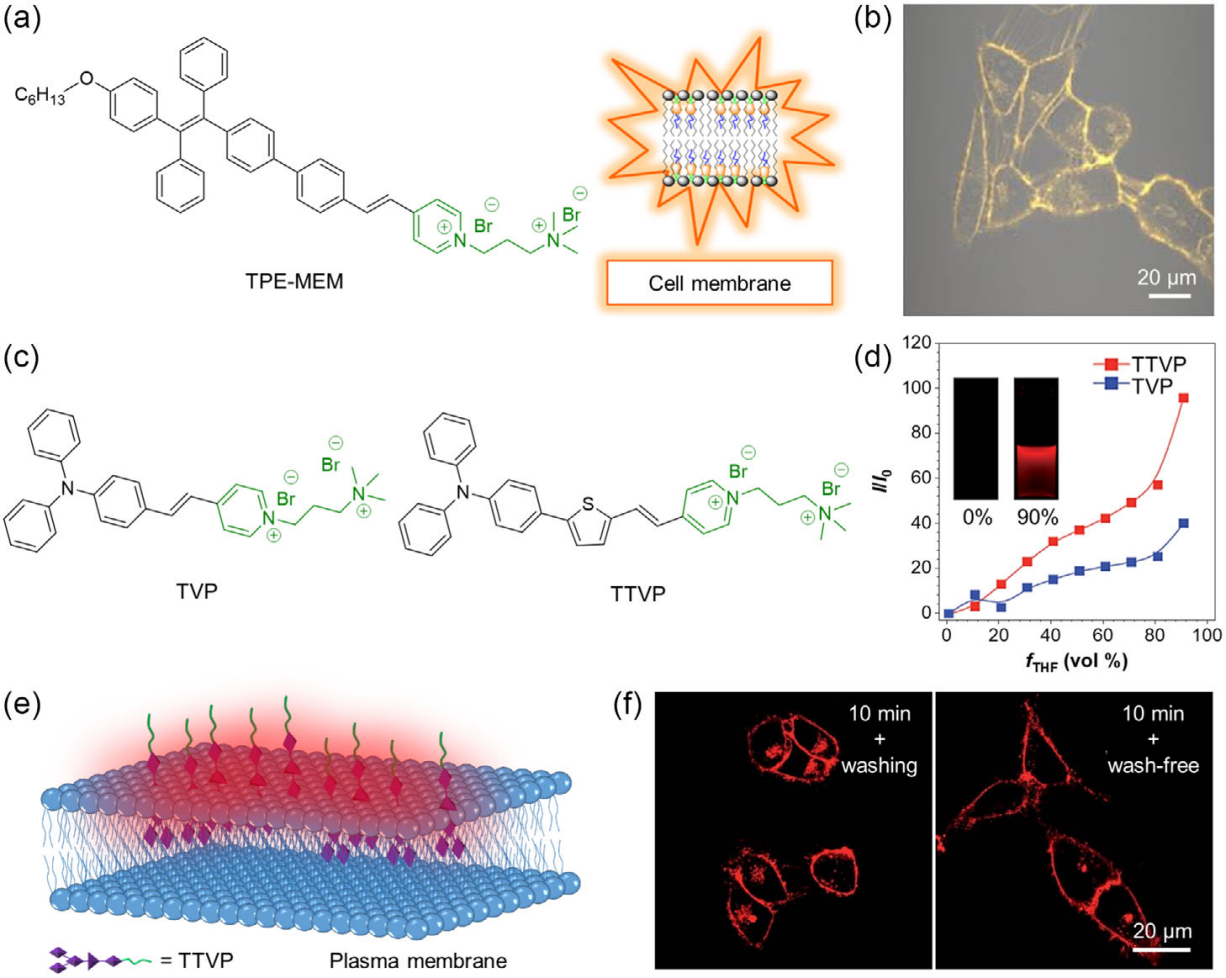
a) Chemical structure of tetraphenylethene (TPE)–MEM and its targeting effect on the cell membrane; b) confocal image of HeLa cells stained with TPE–MEM. b) Reproduced with permission.^[^ ^65^ ^]^ Copyright 2019, American Chemical Society. c) Chemical structures of TVP and TTVP; and d) plots of the relative emission peak intensity (*I*/*I*_0_) of TVP and TTVP versus the composition of the water/THF mixtures (*f*_THF_). Inset: fluorescence (FL) photographs of TTVP in an aqueous solution and in a water/THF mixture (*f*_THF_ = 90%) under 365 nm UV irradiation. e) Schematic illustration of plasma‐membrane‐specific imaging using TTVP; and f) confocal images of living HeLa cells after incubation with TTVP using washing and wash‐free procedures. d,f) Reproduced with permission.^[^ ^66^ ^]^ Copyright 2018, Royal Society of Chemistry.

Organic dyes with positive charges are widely used in biological research. With these positive charges, they can stain negatively charged biomacromolecules such as nucleic acids and proteins in cells. For example, ethidium bromide is a fluorescent dye commonly used in molecular biology for staining nucleic acids, particularly DNA. The cationic nature of EtBr enables strong binding to the negatively charged phosphate backbone of DNA.^[^ ^62^ ^]^ Also, JC‐1 is a positively charged dye used to monitor mitochondrial health through the accumulation in the electronegative interior of the mitochondrion.^[^ ^63^ ^]^ Thus, water‐soluble AIEgens bearing positive charges can visualize negatively charged organelles with their superior emission properties. Zhao et al. found that a tetraphenylethene (TPE) derivative with two hydrophilic amines can intercalate into the membrane of the bacteria and generate an NIR emission peak at about 650 nm. The presence of the positively charged pyridinium salt and quaternary amine endowed the AIEgen with good hydrophilicity to enable wash‐free bacterial imaging and a precise bacterial killing effect using light.^[^ ^64^ ^]^ Based on this work, Zhang et al. further regulated the hydrophilicity of TPE derivatives and generated a membrane‐targeting AIEgen called TPE–MEM (**Figure** **2**a).^[^ ^65^ ^]^ TPE–MEM is a water‐soluble compound that can be used for selective cell‐membrane imaging without washing. This is due to its membrane‐targeting capability and AIE property. As shown in Figure 2b, the membrane structure of HeLa cells was clearly observed in high contrast upon the administration of TPE–MEM. Interestingly, TPE–MEM could efficiently generate ROS under room‐light irradiation. Thus, TPE–MEM was a promising water‐soluble NIR AIEgen to enable a visible observation of cell necrosis and precise phototherapeutic under mild conditions. In 2018, Wang et al. designed and synthesized two water‐soluble NIR AIEgens called TVP and TTVP (Figure 2c).^[^ ^66^ ^]^ Both AIEgens showed emission wavelengths longer than 600 nm, while introducing a thiophene in TTVP further redshifted the emission peak to 700 nm to enable superior NIR imaging. TVP and TTVP showed very weak emissions in water because of their good water solubility. However, in the water/tetrahydrofuran (THF) mixture with a high fraction of THF (*f*_THF_) (a poor solvent for TVP and TTVP), they would emit intense NIR light (Figure 2d). When treating cancer cells with TTVP, the positively charged groups of TTVP enabled a strong binding to negatively charged membrane through electrostatic interactions. The hydrophobic emitting moiety of TTVP would thus be embedded into the hydrophobic region of cell membranes to give evident emission through activating the RIM effect (Figure 2e). It is worth noting that TTVP would not shortly pass through phospholipid bilayers due to its good hydrophilicity, which ensured a membrane‐specific staining behavior. As shown in Figure 2f, the plasma membrane of HeLa cells was visualized in a distinct and fast manner, regardless of the washing or non‐washing process after cell staining. Combined with its efficient ROS generation, TTVP demonstrated excellent performance in precise phototheranostics.

**Figure 3:**
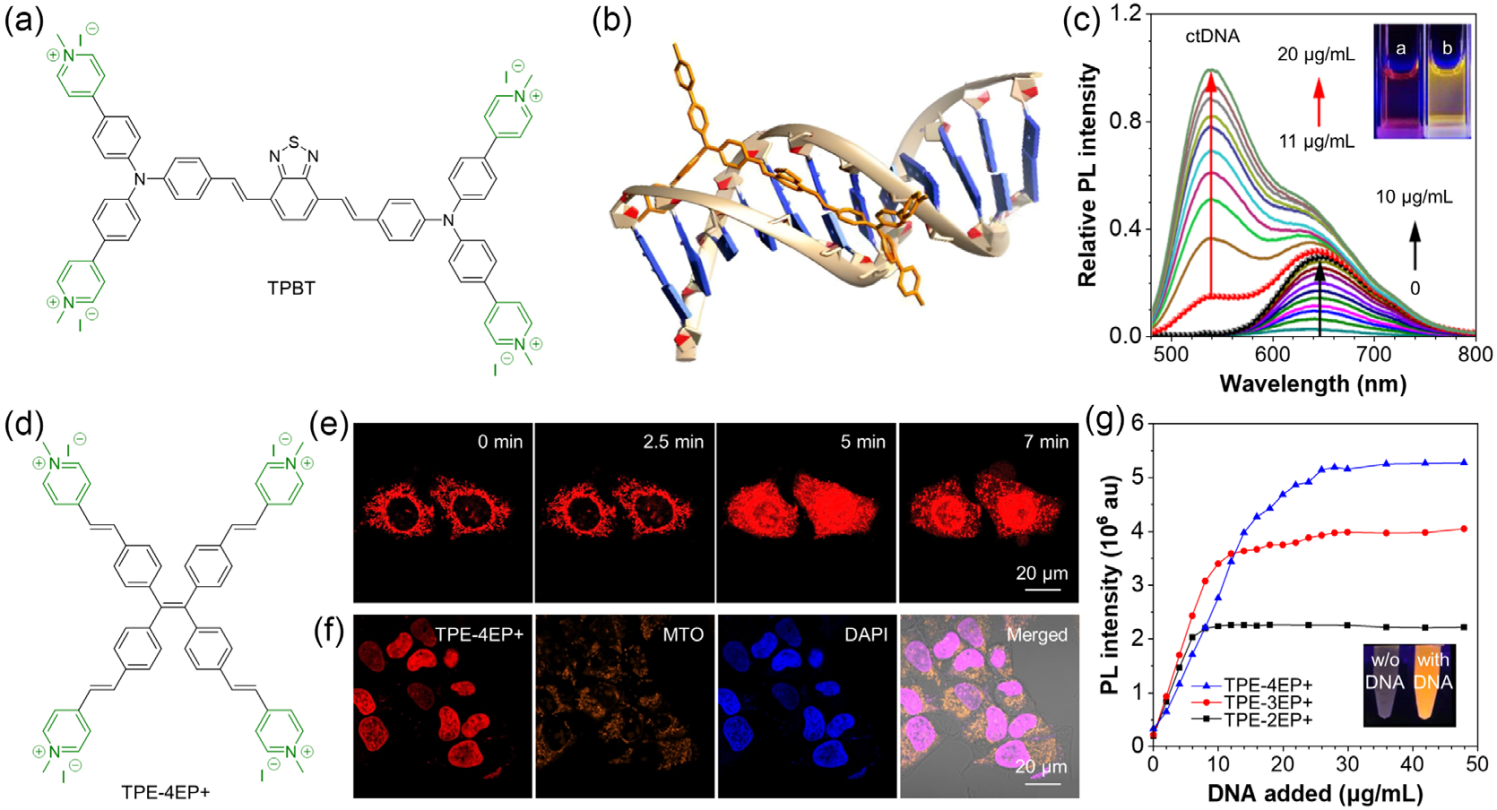
a) Chemical structure of TPBT; b) 3D structure of TPBT and molecular modeling for its interaction with a dodecamer sequence of ctDNA d(CGCGAATTCGCG); and c) photoluminescence (PL) change of an aqueous solution of TPBT (10 μm) with the stepwise addition of ctDNA. Inset: FL photographs of the aqueous solution of TPBT in the presence of: a) 10 μg mL^−1^ or b) 20 μg mL^−1^ ctDNA. b,c) Reproduced with permission.^[^ ^68^ ^]^ Copyright 2019, American Chemical Society. d) Chemical structure of TPE–4EP+; e) real‐time confocal imaging of HeLa cells stained with TPE‐4EP+ (1 μm) under continuous 405 nm laser irradiation; and f) confocal images of fixed HeLa cells stained with TPE–4EP+ (1 μm), MitoTracker orange (MTO) (200 nM), and DAPI (1 μm); and g) plots of PL intensity at 605 nm versus the concentration of added ctDNA. Inset: FL photographs of the TPE–4EP+ aqueous solution in the absence and presence of ctDNA under 365 nm UV irradiation. e–g) Reproduced with permission.^[^ ^69^ ^]^ Copyright 2019 American Chemical Society.

In addition to plasma membranes, water‐soluble NIR AIEgens with positive charges can also target nucleic acids for real‐time monitoring in high quality and fidelity.^[^ ^67^ ^]^ Gao et al. developed an AIEgen called TPBT bearing four positively charged groups to specifically recognize double‐stranded DNA (dsDNA) (**Figure** **3**a).^[^ ^68^ ^]^ Molecular modeling calculations showed that the benzothiadiazole core and the two vinylene bridges on TPBT could perfectly insert into the minor groove of dsDNA (Figure 3b). The strong interaction between TPBT and dsDNA triggered a green FL signal with an emission peak at around 540 nm. Interestingly, TPBT could loosely bind to negatively charged analytes, resulting in an NIR emission at approximately 640 nm, such as dsDNA, ssDNA, proteins, and other polyanionic analytes. As shown in Figure 3c, the FL peak at 645 nm gradually increased as the concentration of existing dsDNA rose from 0 to 10 μg mL^−1^. However, a new emission peak at 537 nm appeared when the dsDNA concentration exceeded the threshold. This green emission was exclusively triggered by dsDNA and is believed to result from the conformational change of TPBT upon groove binding. Thanks to its high sensitivity, TPBT could distinguish single‐nucleotide polymorphisms in DNA sequences and detect UV light‐induced DNA damage. Thus, TPBT offers a robust and facile option for genomic and disease diagnosis. In contrast, Zhang et al. designed a self‐reporting AIE photosensitizer called TPE–4EP+ with four polar pyridinium heads (Figure 3d).^[^ ^69^ ^]^ TPE–4EP+ was not only a fluorescent dye but also capable of inducing the generation of ROS. When introduced to HeLa cells, TPE–4EP+ first stained the mitochondria. After continuous 5 min laser irradiation, translocation of TPE–4EP+ could be clearly observed, and its FL signal mainly appeared in the nucleus (Figure 3e). Annexin V‐FITC/PI staining confirmed that this translocation resulted in cell apoptosis. TPE–4EP+ exhibited high specificity for the nucleus, as evidenced by its excellent co‐localization with 4′,6‐diamidino‐2‐phenylindole (DAPI) (Figure 3f). The authors further validated the nucleus‐targeting effect by adding dsDNA to TPE–4EP+ (Figure 3g). The results proved that TPE–4EP+ bound to dsDNA and emitted bright FL. The binding was stronger for AIEgens with more positive charges, as TPE–4EP+ with four positive charges exhibited more significant FL enhancement than TPE–3EP+ with three positive charges and TPE–2EP+ with two positive charges.

**Figure 4:**
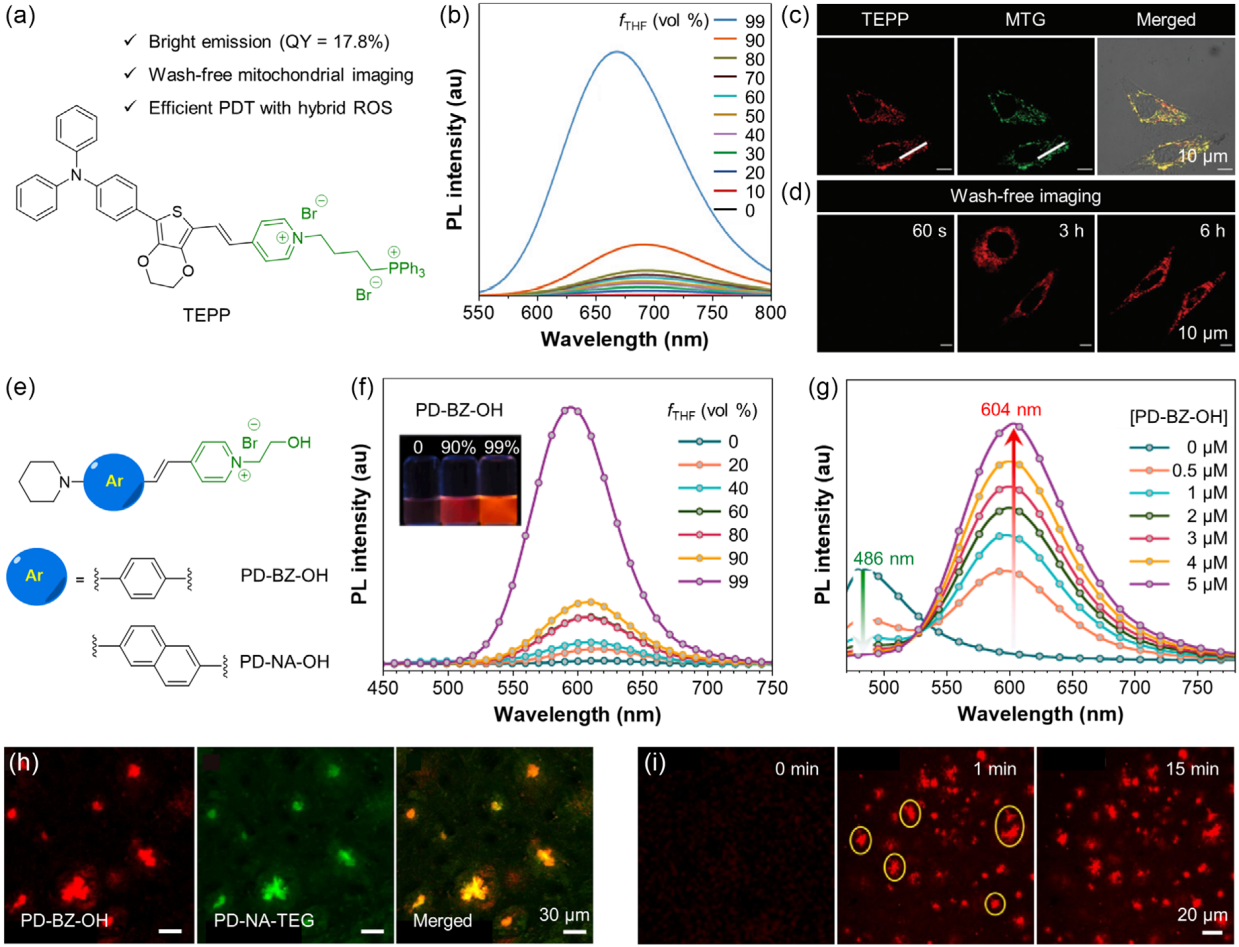
a) Chemical structure and key features of TEPP; b) PL spectra of TEPP in water/THF mixtures with different *f*_THF_; c) confocal images of HeLa cells co‐stained with TEPP and MTG; d) confocal images of HeLa cells stained with TEPP for 60 s, and 3 and 6 h without the washing protocol. b–d) Reproduced with permission.^[^ ^71^ ^]^ Copyright 2022, Royal Society of Chemistry. e) Chemical structures of PD–BZ–OH and PD–NA–OH; f) PL spectra of PD–BZ–OH in water/THF mixtures with different *f*_THF_. Inset: FL photographs of PD–BZ–OH in the water/THF mixtures with *f*_THF_ of 0%, 90%, and 99% under 365 nm irradiation. g) PL spectra of ThT (5 μm) with A*β* fibrils (10 μm) in PBS with the gradual addition of PD–BZ–OH; h) confocal images of A*β* deposits co‐stained with PD–BZ–OH and PD–NA–triethylene glycol (TEG); and i) in situ developing confocal images of A*β* deposits stained by PD–BZ–OH. f–i) Reproduced with permission.^[^ ^72^ ^]^ Copyright 2022, Elsevier B.V.

Mitochondria, the powerhouse of cells, are small subcellular organelles responsible for generating most of the energy for cells. There has been a growing interest in organic molecules that can target mitochondria, as dysfunctions in mitochondria are closely linked to neurological and cardiovascular diseases. Also, mitochondria‐targeting antitumor agents have shown great potential.^[^ ^70^ ^]^ Due to its positive charge nature, triphenylphosphonium is widely used in designing mitochondria‐targeting drugs. The electrostatic interactions with negatively charged mitochondrial membranes enable triphenylphosphonium to target the mitochondria effectively.^[^ ^70^ ^]^ Xu et al. designed a bright water‐soluble AIEgen called TEPP for wash‐free mitochondrial imaging and precise PDT.^[^ ^71^ ^]^ Combining a triphenylamine and a 3,4‐ethylenedioxythiophene (EDOT) as the electron donor with a pyridinium moiety as the electron acceptor through *π*‐conjugation, a typical D–A structure was created for efficient ROS generation (**Figure** **4**a). It is noteworthy that EDOT played multiple roles in the molecular design of TEPP based on its strong hydrophilicity, steric hindrance, and electron‐donating ability. TEPP demonstrated a typical AIE property when the *f*_THF_ of water/THF mixtures increased (Figure 4b). This unique characteristic was particularly helpful for wash‐free imaging. To further illustrate the exceptional mitochondrial specificity of TEPP, confocal images of HeLa cells co‐stained with TEPP and MitoTracker green (MTG) were obtained (Figure 4c). The resulting images demonstrated that TEPP selectively targets mitochondria with a correlation coefficient of 0.89, representing excellent localization accuracy. Then, a wash‐free experiment was performed to evaluate the long‐term imaging ability of TEPP. As shown in Figure 4d, filamentous structures were clearly visualized after 3 or 6 h of incubation, indicative of good mitochondrial specificity, while 60 s of incubation resulted in a very weak FL signal. The superior water solubility of TEPP ensured an FL turn‐on process. TEPP could be well dispersed in the culture medium without emitting any FL unless enriched on mitochondria. Wang et al. designed two water‐soluble AIEgen called PD–BZ–OH and PD–NA–OH for labeling A*β* plaques in a whole brain (Figure 4e).^[^ ^72^ ^]^ PD–BZ–OH showed no emission in its aqueous solution, while a high *f*_THF_ could trigger the FL (Figure 4f). To confirm the affinity of PD–BZ–OH for A*β* fibers, the authors mixed ThT (5 μm) with A*β* fibers (10 μm) to create ThT‐binding A*β* aggregates with a green emission. Upon the addition of PD–BZ–OH (0–5 μm) into the ThT‐binding A*β* aggregates, the green FL of ThT at 486 nm dropped over time, while the red FL of PD–BZ–OH at 604 nm increased (Figure 4g). This result suggested that PD–BZ–OH could occupy the binding sites that originally belonged to ThT due to its higher binding affinity to A*β* fibers. To further study the targeting effect of PD–BZ–OH, brain tissue sections of a transgenic mouse were incubated with PD–BZ–OH and PD–NA–triethylene glycol (TEG), the latter of which is a probe that strongly binds to A*β* plaques with greenish‐yellow emission. Fluorescent co‐localization imaging revealed that the green fluorescent spots emitted by PD–NA–TEG almost completely overlapped with the red fluorescent spots emitted by PD–BZ–OH (Figure 4h), suggesting that PD–BZ–OH was an effective probe for the specific detection of A*β* plaques. Mouse brain tissue sections were then stained with PD–BZ–OH to detect A*β* deposits in real time (Figure 4i). After applying the probe solution (2 μm in phosphate buffered saline (PBS)) to A*β* deposits for 1 min, the emerging red FL signal was observed, indicating the quick staining ability of PD–BZ–OH. The FL intensity remained stable even after 15 min of continuous scanning, proving the outstanding photostability of PD–BZ–OH.

### 3.2 Negatively Charged AIEgens

**Figure 5:**
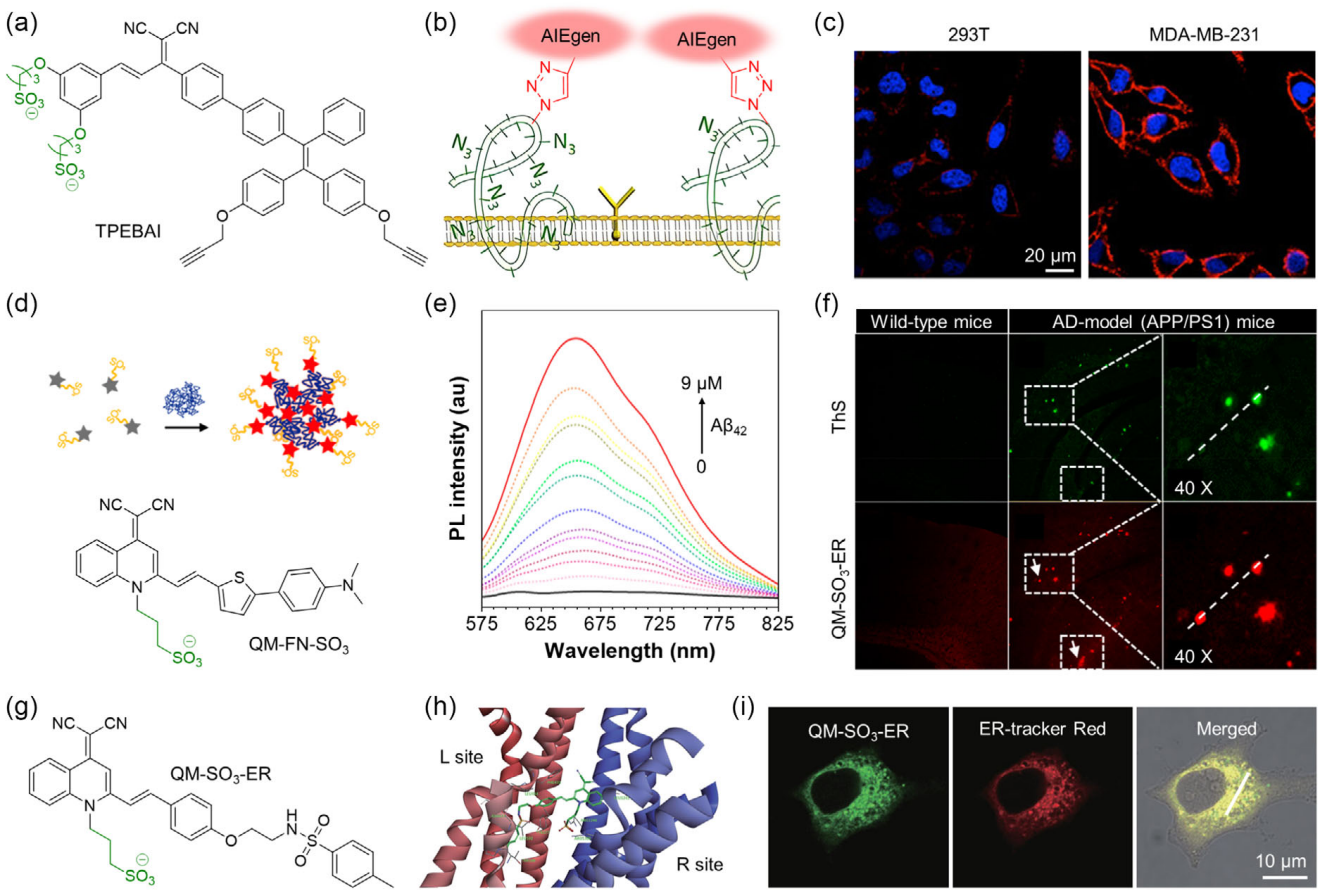
a) Chemical structure of TPEBAI; b) schematic diagram of TPEBAI specifically recognizing cells expressing free Ac_3_ManNAz via click reaction; c) confocal images of MDA–MB‐231 tumor cells and 293T normal cells labeled with TPEBAI (10 μm) after pretreatment with cRGD–S–Ac_3_ManNAz, respectively. b,c) Reproduced with permission.^[^ ^74^ ^]^ Copyright 2018, American Chemical Society. d) Chemical structure of QM–FN–SO_3_ and schematic diagram of A*β* mapping using turn‐on NIR probes; e) PL spectra of QM–FN–SO_3_ with stepwise addition of A*β*_42_ aggregates (0 to 9 μm) in PBS; f) histological staining of the brain slices taken from the hippocampus region of wild‐type mice and Alzheimer's disease model (APP/PS1 transgenic) mice using ThS and QM–FN–SO_3_, respectively. e,f) Reproduced with permission.^[^ ^79^ ^]^ Copyright 2019, American Chemical Society. g) Chemical structure of QM–SO_3_–ER; h) molecular modeling of QM–SO_3_–ER binding to the K_ATP_ channel (PDB: 6BAA) in the subunit of sulfonylurea receptor 1 (SUR1); and i) confocal images of HeLa cells co‐stained with QM–SO_3_–ER and ER‐tracker Red. h,i) Reproduced under the terms of the CC‐BY Creative Commons Attribution 4.0 International license (https://creativecommons.org/licenses/by/4.0)^[^ ^80^ ^]^ Copyright 2020, The Authors, published by Oxford University Press on behalf of China Science Publishing & Media Ltd.

Organic dyes with negative charges are commonly used for FL imaging. For example, Alexa Fluor dyes are a big family of negatively charged and hydrophilic fluorescent dyes frequently used in FL microscopy. All Alexa Fluor dyes are synthesized through sulfonation of their parental fluorophores like fluorescein, coumarin, cyanine, or rhodamine.^[^ ^73^ ^]^ Decorating NIR AIEgens with sulfonate groups can also endow them with water solubility for wash‐free bioimaging. Hu et al. synthesized a water‐soluble light‐up AIE probe called TPEBAI (**Figure** **5**a).^[^ ^74^ ^]^ The authors developed a metabolic precursor to generate glycans with azide groups on the cancer cell membrane with high selectivity. The FL signal from the probe would be activated upon a click reaction with azide groups on the surface of cancer cells to enable selective cancer cell imaging with a low background signal (Figure 5b). As shown in Figure 5c, 293T cells without azide expression showed weak FL intensity, while the FL intensity of MDA–MB‐231 cells was much stronger due to the high expression of azide groups. Additionally, fluorescent dyes with sulfonate groups can effectively bind to biomarkers. For instance, indocyanine green (ICG), a clinically used fluorescent dye with sulfonate groups, binds to plasma proteins to significantly enhance its brightness upon injection into the bloodstream.^[^ ^75^ ^]^ Another study has shown that the chemical modification of sulfonate groups on CH1055, a carboxylated NIR‐II dye, can increase its brightness through supramolecular binding to serum proteins.^[^ ^76^ ^]^ Based on these studies, sulfonation of AIEgens can provide a useful strategy for developing smart AIE probes.^[^ ^77^ ^]^ Sun et al. utilized a sulfonate‐containing zwitterionic structure to modulate the FL property of AIEgens in blood. The NIR FL from the aggregates could be turned on through the strong interaction with low‐density lipoprotein to achieve the rapid diagnosis of hyperlipidemia patients.^[^ ^78^ ^]^ Thus, water‐soluble NIR AIEgens with sulfonate groups can serve as sensitive probes for the detection of biomarkers due to their low background and targeting ability. Fu et al. designed and synthesized an NIR AIE probe called QM–fibronectin (FN)–SO_3_ for in situ mapping of A*β* plaques (Figure 5d).^[^ ^79^ ^]^ The AIE building block, quinoline–malononitrile (QM), was used to overcome the quenching effect, while sulfonate groups were introduced to enhance the hydrophilicity. QM–FN–SO_3_ had a low background signal in an aqueous solution, while adding A*β*_42_ would efficiently turn on its FL (Figure 5e). To evaluate whether QM–FN–SO_3_ could be an alternative to commercial probes, the authors conducted in vitro fluorescent staining of A*β* plaques of brain tissue slices from Alzheimer's disease–model (APP/PS1 transgenic) mice and wild‐type mice (Figure 5f). The results showed that the commercial dye called ThS and QM–FN–SO_3_ had almost identical staining results, indicating that QM can effectively image A*β*_42_ plaques. In particular, QM–FN–SO_3_ yielded more abundant feedback than ThS, which proved the high fidelity of bioimaging using QM–FN–SO_3_. To explore more possibilities of using QM to construct AIE probes, Zhu et al. decorated the hydrophilic QM structure with an endoplasmic reticulum (ER)‐targeting *p*‐toluenesulfonamide group (Figure 5g).^[^ ^80^ ^]^ The resulting QM–SO_3_–ER dispersed well in water with an undetectable hydrodynamic diameter, ensuring a low FL background. Molecular docking studies showed that QM–SO_3_–ER could specifically bind to the subunit of sulfonylurea receptor 1 (SUR1) of the K_ATP_ channel on the ER membrane (Figure 5h). The authors then evaluated the performance of QM–SO_3_–ER as an ER‐sensing probe. As demonstrated in HeLa cells stained by ER‐tracker Red, QM–SO_3_–ER exhibited excellent ER‐targeting ability with a high Pearson's correlation coefficient of 0.9520 (Figure 5i).

## 4 AIEgens with Hydrophilic Chains

**Figure 6:**
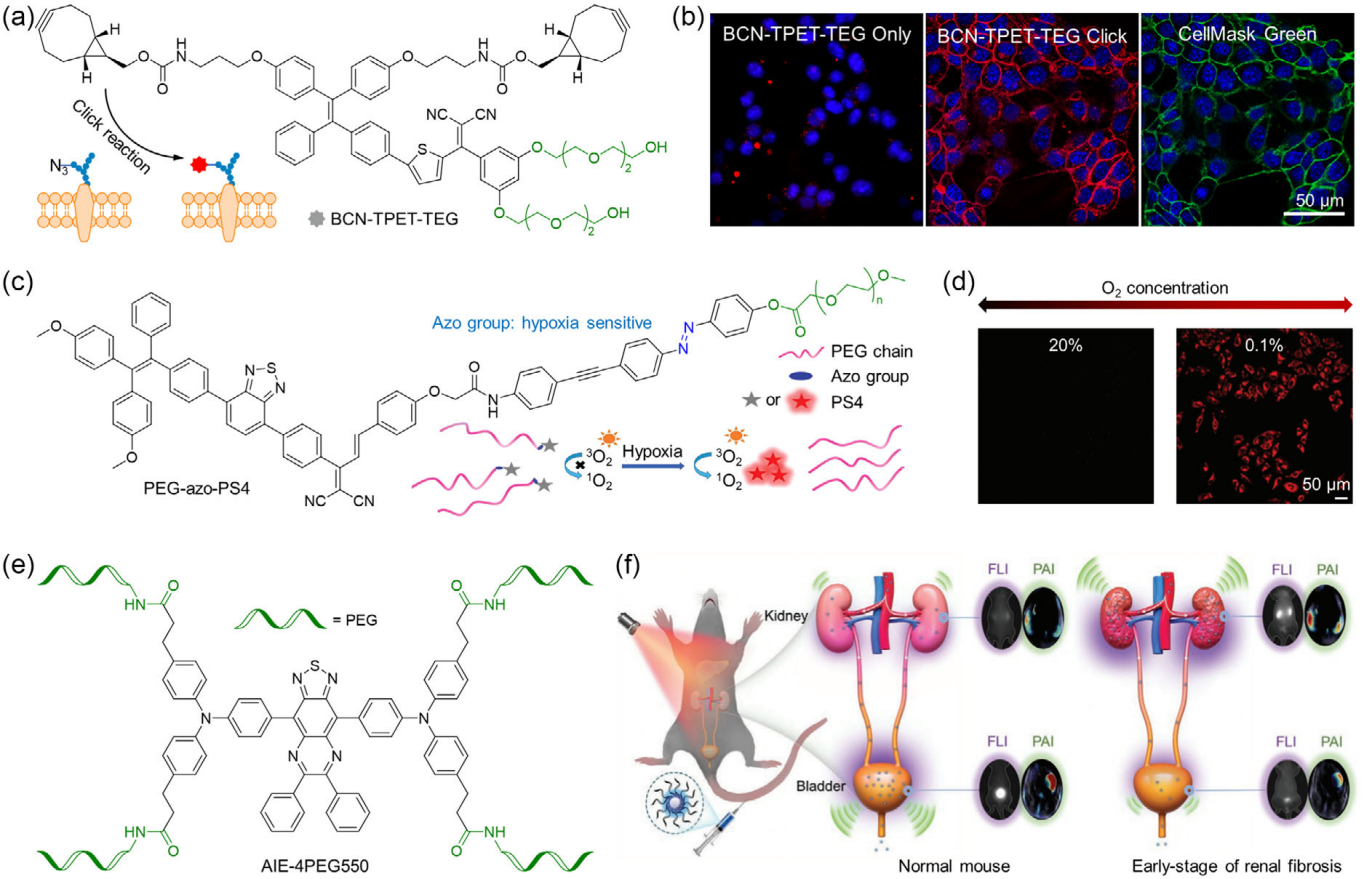
a) Chemical structure of bicyclo[6.1.0]nonyne (BCN)–TPET–TEG and schematic diagram of its specific recognition of AzAcSA‐pretreated tumor cells via copper‐free click reaction; b) confocal images of 4T1 cells treated with BCN–TPET–TEG (5 μg mL^−1^) only, BCN–TPET–TEG (5 μg mL^−1^) bioorthogonal labeling, and CellMask Green (5 μm) treatment, respectively. b) Reproduced with permission.^[^ ^84^ ^]^ Copyright 2018, Wiley‐VCH. c) Chemical structure of PEG–azo–PS4 and schematic diagram of its hypoxia‐mediated FL and photosensitization enhancement; d) confocal images of HepG‐2 cells after treatment with PEG–azo–PS4 (5 μm) at different oxygen levels. d) Reproduced with permission.^[^ ^85^ ^]^ Copyright 2021, Royal Society of Chemistry. e) Chemical structure of AIE‐4PEG550; and f) schematic diagram of the application of AIE‐4PEG550 on FL/PA bimodal imaging of renal fibrosis progress in mice. f) Reproduced with permission.^[^ ^42^ ^]^ Copyright 2022, Wiley‐VCH.

AIEgens with hydrophilic chains are an emerging class of water‐soluble AIEgens for biological applications. Hydrophilic chains like PEG and OEG show good water solubility because they have many ether linkages (–O–) that can form hydrogen bonds with water molecules. Chemical modification with hydrophilic chains has been widely used for organic electronic devices and molecular optical agents.^[^ ^81^, ^82^, ^83^ ^]^ Compared to the introduction of charged groups, modifying organic molecules with hydrophilic chains may have higher stability and biosafety in physiological conditions because they would be less influenced by ionic strength, pH, and other biomolecules. Hu et al. developed a light‐up AIE probe called bicyclo[6.1.0]nonyne (BCN)–TPET–TEG for in vivo bioorthogonal tumor labeling (**Figure** **6**a).^[^ ^84^ ^]^ The hydrophilic TEG rendered BCN–TPET–TEG sufficient hydrophilicity and inertness to normal tissues in mice. The BCN moiety enabled copper‐free click reactions with azide groups on tumor cells, resulting in FL illumination owing to the RIM effect (Figure 6a). Upon incubation with BCN–TPET–TEG (5 mg mL^−1^), azide‐free 4T1 cells did not show any FL signal, suggesting the low FL background of BCN–TPET–TEG in the culture medium (Figure 6b). However, when 4T1 cells expressing azide groups were treated with BCN–TPET–TEG, it could lead to the generation of a strong NIR FL signal, which overlapped well with the green FL signal of the cell membrane tracker (Figure 6b). Li et al. reported a novel PEGylated AIE‐active molecular probe for hypoxic tumor imaging and PDT.^[^ ^85^ ^]^ After demonstrating the singlet oxygen generation capacity of the AIE photosensitizer (PS4), the authors modified PS4 with a PEG chain (approximately 2.0 kD) via an azo group (Figure 6c). Under hypoxic conditions, a significant FL increase could be observed due to the cleavage of the hypoxia‐responsive azo group. The released product exhibited robust singlet oxygen generation, indicating that both FL and PDT were activated under hypoxia. Figure 6d showed a prominent red emission when HepG2 cells were incubated with PEG–azo–PS4 under hypoxic conditions (Figure 6d). This result evidenced the effectiveness of the design strategy of hypoxia‐responsive water‐soluble AIEgens.

Modification with hydrophilic chains can also influence the pharmacokinetic properties of optical agents.^[^ ^82^, ^86^ ^]^ Currently, many optical imaging probes are cleared by the reticuloendothelial system. Excessive accumulation of probes in the hepatobiliary clearance pathway (liver and spleen) may result in potential organ toxicity. In contrast to other agents, renally clearable optical agents can be efficiently filtered through the kidneys and eliminated from the body with minimal metabolism, thereby ensuring a high biosafety level. Moreover, renally clearable optical agents are intrinsically suitable for targeted kidney imaging due to their renal excretion. The imaging mechanism includes passive monitoring of the glomerular filtration rate and detection of early kidney injury biomarkers. The development of AIE‐active renal‐clearable optical agents is promising for detecting kidney diseases. Yan et al. designed a nanosized fluorophore named AIE‐4PEG550 for longitudinal visualization of early‐stage fibrosis progression through short‐wave infrared (900–1700 nm) FL and photoacoustic (PA) bimodal imaging (Figure 6e).^[^ ^42^ ^]^ By injecting AIE‐4PEG550 into mice with normal renal function or varying degrees of renal fibrosis, the distinction in FL and PA signals at kidney and bladder sites enabled the identification of the fibrosis stage (Figure 6f). Due to kidney dysfunction and slowed metabolism via urinary excretion, mice with early‐stage renal fibrosis showed a higher FL/PA intensity ratio between the kidney and bladder than normal mice. This work provided a facile probe for real‐time nonintrusive assessment of renal fibrosis in living mice.

## 5 AIEgens with Bioactive Moieties

**Figure 7:**
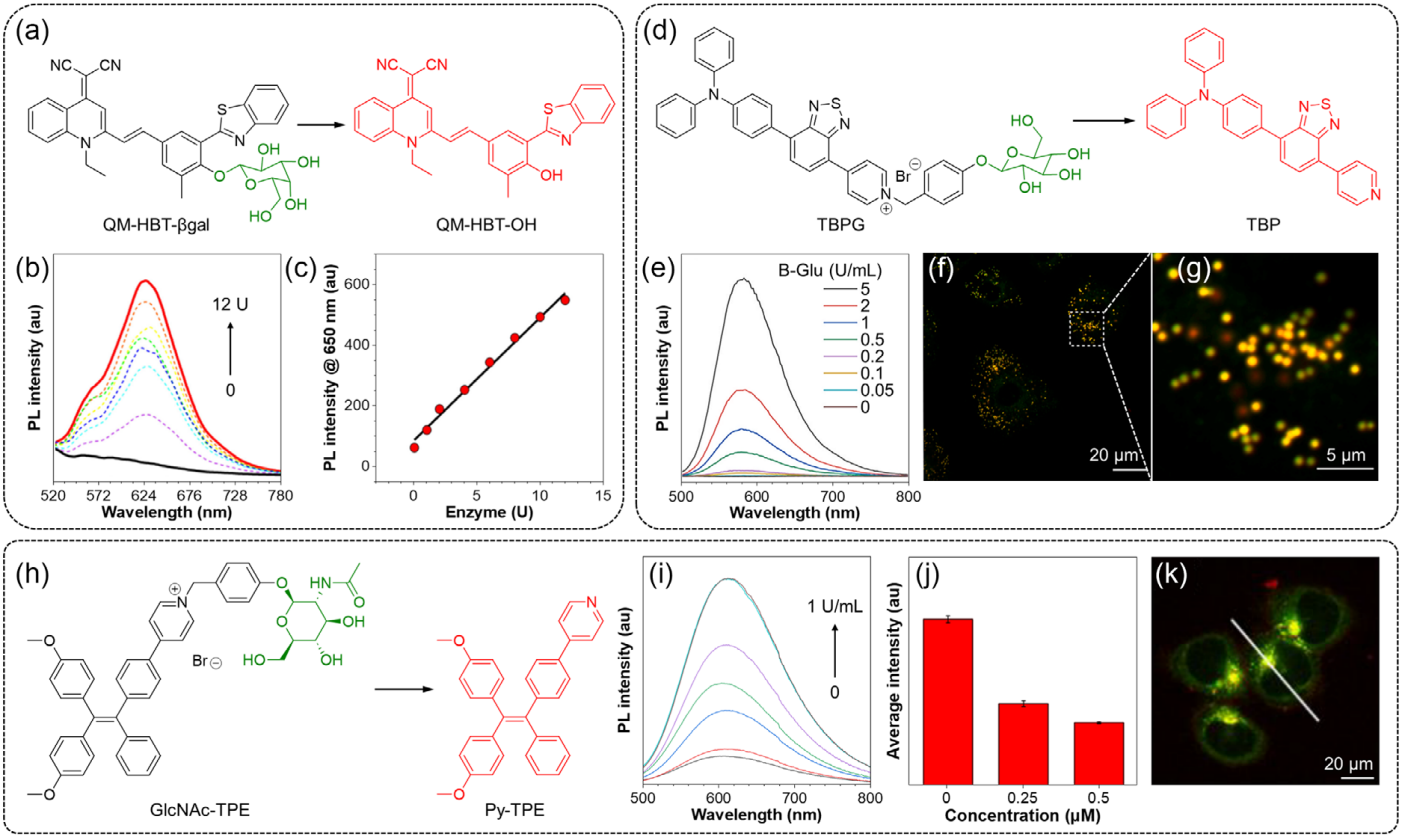
a) Chemical structure of QM–HBT–*β*gal and its mechanism of *β*‐galactosidase (*β*‐gal) detection; b) PL spectra of QM–HBT–βgal (10 μm) upon treatment with increasing concentrations of *β*‐gal (0–12 U) after 6 h incubation; c) PL intensity of QM–HBT–*β*gal at 650 nm as a function of *β*‐gal concentration after 6 h incubation. b,c) Reproduced under the terms of the CC‐BY Creative Commons Attribution 4.0 International license (https://creativecommons.org/licenses/by/4.0)^[^ ^88^ ^]^ Copyright 2019, The Authors, published by Frontiers. d) Chemical structure of TBPG and its mechanism of *β*‐Glu detection; e) PL spectra of TBPG (50 μm) upon treatment with increasing concentrations of *β*‐Glu (0–5 U mL^−1^) after 1 h incubation in McIlvaine buffer; f,g) confocal image and its enlarged view of A549 cells co‐stained by LipidSpot 610 and TBPG for 60 min. e–g) Reproduced with permission.^[^ ^89^ ^]^ Copyright 2023, Elsevier Ltd.; h) chemical structure of GlcNac‐TPE and its mechanism of *β*‐*N*‐acetylhexosaminidase (Hex) detection; i) PL spectra of GlcNAc–TPE (10 μm) upon the addition of Hex (0–1 U mL^−1^) at 37 °C for 60 min; j) average intensity of cells stained with GlcNAc–TPE in the absence and presence of PUGNAc; and k) confocal image of HCT116 cells co‐stained with GlcNAc–TPE and LysoTracker Green DND‐26. i–k) Reproduced with permission.^[^ ^90^ ^]^ Copyright 2019, American Chemical Society.

AIEgens with bioactive moieties have emerged as a promising class of water‐soluble AIEgens for biological applications. Incorporating bioactive moieties such as peptides, sugars, or nucleic acids can endow the AIEgens with superior hydrophilicity and specific biological functions. Yuan et al. developed a theranostic Pt(IV) prodrug for targeted drug delivery and real‐time evaluation of its therapeutic response in situ.^[^ ^87^ ^]^ The prodrug contained a cyclic arginine–glycine–aspartic acid (cRGD) tripeptide that targets integrin αvβ3 overexpressed cancer cells, a green‐emissive AIE apoptosis sensor tetraphenylsilole (TPS), and a caspase‐3 substrate Asp–Glu–Val–Asp (DEVD) peptide. Upon cellular uptake, the prodrug was reduced to the active Pt(II) drug to induce cell apoptosis, which further activated caspase‐3 to cleave the DEVD sequence and liberate the hydrophobic TPS moiety. The FL signal generated by the aggregation of TPS enabled early observation of its therapeutic response in cells with a high signal‐to‐noise ratio. Fu et al. reported a novel enzyme‐responsive NIR AIE probe called QM–HBT–βgal for tracking endogenous *β*‐galactosidase (*β*‐gal) activity in living cells (**Figure** **7**a).^[^ ^88^ ^]^ QM–HBT–*β*gal was almost non‐emissive in aqueous media. However, upon the addition of *β*‐gal, the masking groups at the hydroxyl moieties of QM–HBT–*β*gal were removed to trigger the aggregation of hydrophobic QM–HBT–OH molecules, and then the strong NIR FL could be detected (Figure 7b). The experimental results showed that the FL intensity at 650 nm increased gradually with increasing *β*‐gal concentration (0–12 U). Moreover, a linear correlation was observed between the FL intensity and the concentration of *β*‐gal (Figure 7c). Yao et al. developed a water‐soluble NIR AIEgen called TBPG for the detection of *β*‐glucosidase (*β*‐Glu) (Figure 7d).^[^ ^89^ ^]^ Upon hydrolysis of TBPG by *β*‐Glu, the FL from the generated TBP allowed sensitive and selective detection of *β*‐Glu (Figure 7e). Figure 7f and 6g illustrate the overlap of confocal images stained by LipidSpot 610 and TBPG. The significant overlap suggested that the hydrolyzed product of TBPG possesses a remarkable targeting capability for lipid droplets. TBPG was successfully applied to the enzyme analysis of real soil samples. Using a similar pyridinium strategy, Wang et al. synthesized GlcNAc–TPE for in situ and real‐time visualization of *β*‐N‐acetylhexosaminidase (Hex) in the lysosome, which has been reported to possess vital physiological functions (Figure 7h).^[^ ^90^ ^]^ FL titration experiments demonstrated that the FL intensity increased upon the gradual addition of Hex from 0 to 1 U mL^−1^ into the GlcNAc–TPE solution, indicative of its conversion into Py–TPE (Figure 7i). To further prove that Hex selectively turned on the emission of GlcNAc–TPE, inhibition experiments were conducted with *O*‐(2‐acetamido‐2‐deoxy‐_D_‐glucopyranosylidene)amino‐*N*‐phenyl‐carbamate (PUGNAc), a commonly used inhibitor of Hex (Figure 7j). Upon treatment with PUGNAc, the bright red emission in HCT116 cells exhibited a sharp decrease, suggesting that the hydrolysis of GlcNAc‐TPE catalyzed by Hex was responsible for the detected FL signal. Confocal images of HCT116 cells revealed that the bright red emission of Py‐TPE overlapped well with the green FL of LysoTracker Green (Figure 7k). This result indicated that Py–TPE could selectively target lysosomes in live cells, which is crucial for clarifying the physiological roles of Hex in lysosomes.

**Figure 8:**
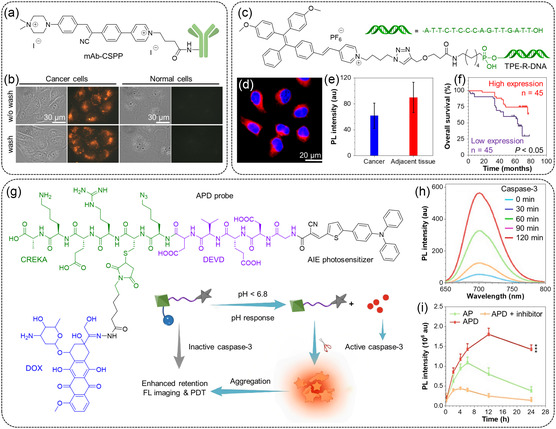
a) Chemical structure of mAb–CSPP; b) bright‐field and fluorescence images of HCC827 cancer cells and COS‐7 normal cells incubated with 10 μg mL^−1^ of mAb–CSPP for 12 h. Images were taken in the probe medium with or without washing protocol. b) Reproduced with permission.^[^ ^92^ ^]^ Copyright 2017, Royal Society of Chemistry. c) Chemical structure of TPE–R–DNA; d) confocal image of mRNA in MCF‐7 cells treated with 10 μg mL^−1^ lipopolysaccharide (LPS) for 2 h and then incubated with TPE–R–DNA (6.6 μm) and Exo III (1.33 U mL^−1^) for 1 h under the standard cell culture conditions; e) PL intensity of renal cancer and its adjacent tissue; f) Kaplan–Meier survival curves of patients categorized into high‐ and low‐expression groups based on the MnSOD mRNA signature. d–f) Reproduced with permission.^[^ ^94^ ^]^ Copyright 2018, American Chemical Society. g) Chemical structure of AIE–Pep–DOX (APD) and schematic diagram of the pH‐responsive release of DOX and the caspase‐3‐responsive aggregation of AIEgen for enhanced tumor theranostics. APD is composed of an NIR AIE photosensitizer, a caspase‐3 specific peptide named DEVD (purple), an FN‐targeting peptide called CREKA (green), and DOX (blue); h) PL spectra of free APD (10 μm, grey line), and APD (10 μm) incubated with caspase‐3 (5 U mL^−1^) for 30, 60, 90, and 120 min; and i) change of FL intensity in the tumor region of mice treated with AP, APD, or both APD and inhibitor. h,i) Reproduced with permission.^[^ ^95^ ^]^ Copyright 2022, Elsevier Ltd.

Monoclonal antibodies (mAbs) are critical tools for cancer detection, diagnosis, and treatment. Conjugating mAbs with fluorescent dyes could be a highly effective approach for designing specific molecular imaging probes based on the exceptional target specificity of antibodies.^[^ ^91^ ^]^ Shi et al. described a turn‐on AIE probe called mAb–CSPP, an antibody–AIEgen conjugate, for specific cancer cell imaging in a wash‐free manner (**Figure** **8**a).^[^ ^92^ ^]^ The resulting probe exhibited several advantages, such as low background signal, long‐term intracellular retention, large Stokes shift, and good photostability. In both washing and wash‐free protocols, mAb–CSPP was emissive only in specific cancer cells, while it showed almost no emission in normal cells (Figure 8b). This technique had potential applications in precise cancer diagnosis and antitumor therapy. Deoxyribonucleic acid (DNA) is also a bioactive moiety with satisfactory solubility and biocompatibility. Conjugating AIE fluorophores with functionalized DNA strands ensures promising detection and imaging outcomes.^[^ ^93^ ^]^ Wang et al. designed a DNA‐conjugated amphiphilic AIE probe, TPE–R–DNA, for cancer tissue imaging and prognosis analysis (Figure 8c).^[^ ^94^ ^]^ Due to its high water solubility, TPE–R–DNA had low emission in the absence of manganese superoxide dismutase (MnSOD) mRNA. However, upon adding MnSOD mRNA, TPE–R–DNA was hydrolyzed by exonuclease III (Exo III) to produce the hydrophobic fluorophore. The formed aggregates would result in a dramatic increase in FL signal, and the detection limit of the proposed method toward mRNA was as low as 0.6 pM. As displayed in Figure 8d, the probe emitted strong red FL in cells treated with lipopolysaccharide (LPS), indicating a high intracellular MnSOD mRNA expression level. Then, the authors employed TPE–R–DNA to detect the MnSOD mRNA in cancer tissue. It was found that renal cancer showed reduced MnSOD mRNA expression than the adjacent tissue of renal cancer (Figure 8e). Additionally, the expression level was analyzed to predict outcomes for cancer patients. The results revealed that the MnSOD mRNA expression of patients was positively related to the treatment efficacy (Figure 8f). Therefore, TPE–R–DNA holds great promise in cancer imaging and prognosis assessment. In addition to being a trigger or targeting group, some bioactive moieties can be utilized as a smart linker of different functional moieties to achieve multi‐stimuli‐responsive theranostics. As mentioned earlier, DEVD is a caspase‐3‐specific peptide.^[^ ^87^ ^]^ Yao et al. employed DEVD as a linker for the chemical conjugation of an NIR AIE photosensitizer and a FN‐targeting peptide named Cys–Arg–Glu–Lys–Ala (CREKA). Doxorubicin (DOX) was then linked to CREKA using a pH‐responsive hydrazone bond (Figure 8g).^[^ ^95^ ^]^ The CREKA peptide enabled enhanced tumor‐targeting and cellular internalization through FN recognition. This unimolecular prodrug AIE–Pep–DOX (APD) (AIEgen + DEVD + CREKA + DOX) would show negligible FL and phototoxicity due to the vivid molecular motions when molecularly dissolved in water. However, the hydrazone bond would be cleaved to release DOX to induce cell apoptosis in an acidic tumor microenvironment. Caspase‐3 would thus be activated to cleave DEVD and liberate the hydrophobic AIEgen selectively. This process further led to the aggregation of AIEgens and amplified their FL intensity and PDT activity. The addition of caspase‐3 enabled a turn‐on FL around 700 nm with a 100‐fold increase in intensity at 120 min (Figure 8h). FL imaging was performed in mice bearing 4T1 tumors to study the performance of APD in vivo (Figure 8i). After 24 h, a strong FL signal was observed only in the APD‐treated tumor, while very weak FL was detected in control groups treated with AP (AIEgen + DEVD + CREKA peptide) or APD + FN inhibitor. The aggregation of AIEgens prolonged their tumor retention for long‐term FL imaging and repeatable PDT by only one single‐dose injection. In the following study, the combination of repeatable PDT with immune checkpoint blockade therapy effectively suppressed tumor growth and pulmonary metastasis.

## 6 Conclusion

FL technology using organic fluorophores has become an essential tool for visualizing biological processes with high sensitivity and specificity. NIR AIEgens are regarded as ideal candidates for phototheranostics, which offer a promising solution to overcome the limitation of organic dyes in physiological environments. Water solubility is critical to achieve an outstanding performance of NIR AIEgens in physiological environments, and thus researchers have been dedicated to developing water‐soluble NIR AIEgens. From this perspective, we discussed their design strategies and advanced applications in phototheranostics. The development of water‐soluble NIR AIEgens has made some progress, which is summarized in **Table** **1**. However, this field is still in its infancy with significant challenges and opportunities, which are detailed in the following content, divided into four parts. 1) Smart use of hydrophilic groups: it should be noted that rational choice of hydrophilic groups and targeting groups is vitally important for a successful molecular design. There are several considerations for the rational design of hydrophilic moieties. One consideration is that hydrophilic groups significantly impact the water solubility of probes, which determines how much substance can be effectively absorbed and utilized by the organism after administration. Excellent bioavailability is particularly important for both diagnostic and therapeutic procedures. The AIE‐active cores with NIR emission vary considerably in chemical conjugation and hydrophobicity. Good hydrophilic groups are required for some highly hydrophobic NIR AIE cores to prepare their water‐soluble counterparts.^[^ ^96^ ^]^ In contrast, excessive hydrophilicity may not be conducive to biological applications.^[^ ^97^ ^]^ For example, oseltamivir carboxylate is a drug that selectively inhibits the neuraminidase of influenza viruses A and B. However, because the carboxylate structure is too hydrophilic, its oral bioavailability is only 5% in humans.^[^ ^98^ ^]^ In clinical usage, its ethyl ester form (Tamiflu) is more lipophilic and can be absorbed rapidly after oral administration, with at least 80% of the dose reaching systemic circulation. After absorption, oseltamivir is converted to oseltamivir carboxylate and ethanol mainly by carboxylesterase‐1 in the liver. Therefore, striking an appropriate balance between hydrophilicity and hydrophobicity is essential for optimizing the bioactivity of AIEgens. The second consideration is that the intrinsic amphiphilicity of water‐soluble NIR AIEgens ultimately determines their photophysical properties and performance in phototheranostics. For instance, after introducing hydrophilic moieties, the resulting AIE probes may show different emission properties. If hydrophilic moieties make AIE probes molecularly dissolved in water, both imaging and therapy processes can operate in a turn‐on fashion once activated. In contrast, excessive hydrophobicity of the selected AIE core can induce self‐assembly of AIE probes to form nanoparticles with superior FL and potential phototherapy effects. Moreover, ensuring good photophysical properties and bioactivities can be dilemmatic when introducing hydrophilic moieties into AIE probes. They may show superior sensing performance but weak FL due to the quenching effect of water, while others may show bright emission but a very poor detection capability. The third consideration is to ensure the biosafety of AIE probes. Hydrophilic groups bearing positive charges are widely used in the design of water‐soluble NIR AIEgens. However, some structures can be seriously or potentially toxic. For instance, the structure of quaternary ammonium salt can be widely found in disinfectants, indicating its toxicity.^[^ ^99^ ^]^ Additionally, as mentioned before, EtBr is highly toxic as a mutagen because of its strong binding with DNA. Therefore, researchers should avoid introducing excessive positive charges into water‐soluble NIR AIEgens. Furthermore, it is imperative to conduct exhaustive research on potential long‐term toxicity. 2) Development of water‐soluble NIR‐II AIE probes: more research should be put into designing water‐soluble NIR‐II AIE probes with excitation wavelength in the NIR‐II window since it can improve light penetration for deeper tissue imaging. In general, the synthesis of NIR‐II AIEgens is highly intricate. The further chemical modification of these molecules is even more challenging. For instance, since the commonly used benzobisthiadiazole core is not alkali resistant, the synthetic route to water‐soluble benzobisthiadiazole‐cored AIEgens should be carefully planned prior to practical laboratory synthesis. Furthermore, the selection of appropriate hydrophilic functional groups for NIR‐II AIEgens is particularly demanded, given their intrinsic hydrophobicity. Currently, some NIR‐II AIEgens have been reported with promising results in biosensing.^[^ ^100^, ^101^ ^]^ However, further consideration to improve their water solubility is still highly desired. 3) Preparation of water‐soluble NIR AIEgens with diverse responsibilities: the exploration of responsive moieties to other analytes, such as metal ions, is needed to diversify the application scenarios of water‐soluble NIR AIEgens. Some of metal ions (Na^+^, K^+^, Mg^2+^, Ca^2+^, Zn^2+^, Cu^2+^, Fe^2+/3+^, etc.) are essential for biological processes in living organisms such as communication between cells, DNA regulation, and nerve function, while some others ((Hg^2+^, Cd^2+^, As^3+^, Pb^2+^ Cr^3+/6+^, Ni^2+^, Co^2+^, etc.) are nonessential or even highly toxic.^[^ ^102^ ^]^ As AIE probes with short‐emission wavelengths have shown great potential in metal‐ion detection,^[^ ^102^ ^]^ further development of water‐soluble NIR AIE probes can broaden their applications to sensing metal ions in deep tissues. 4) Construction of multi‐stimuli‐responsive theranostic systems: currently, most probes are single‐responsive. However, due to the complex biological milieu, this molecular design strategy can still produce “false positive” signals.^[^ ^103^ ^]^ This is because undesired target sites may have moderate concentrations of biomarkers, and the AIE probe can be nonspecifically activated during transit. The nonspecificity can result in a large amount of noise and cause serious off‐target effects in phototherapy.^[^ ^104^ ^]^ In addition, single‐locked probes cannot recognize two interlinked biomarkers or molecular events in targeted or disease sites. They only provide limited information about biological processes. In contrast, unimolecular multi‐stimuli‐responsive theranostic agents demonstrate the potential to overcome many of the disadvantages of single‐responsive materials.^[^ ^103^, ^105^ ^]^ They display significantly reduced signal crosstalk and increased spatial resolution, resulting in more precise phototheranostics. Moreover, the ability to provide a response only when specific biomarkers are presented in a particular sequence can be essential in the accurate monitoring of living organisms. This produces specific signal output information as a result of intelligent recognition. And, 5) application expansion of water‐soluble NIR AIEgens: the applications of water‐soluble NIR AIEgen should be expanded and diversified. For example, these materials can be used to detect specific environmental substances for real‐time monitoring or biotoxicity assessment of pollutants. While some AIEgens have been used in this field, water‐soluble NIR AIEgens are supposed to be excellent alternatives due to their advantages in reducing signal interference from other substances to improve signal fidelity.^[^ ^106^, ^107^ ^]^ Suitable water solubility may make NIR AIEgens more sensitive to substances and more suitable for in vivo monitoring with reduced toxicity, given the low concentration of environmental pollutants. Further study is needed to coordinate the roles of luminescent properties, water solubility, and sensitivity in the molecular design of NIR AIE probes.

**Table 1:** A summary of reported water‐soluble NIR AIEgens

AIEgen	Hydrophilic moiety	*λ*_abs_ [nm]a)	*λ*_em_ [nm]b)	Targeting site or analyte	Application	Ref.
TPE–MEM	Pyridinium and quaternary ammonium	395	625	Plasma membrane	Cell membrane imaging and PDT	[65]
TVP	Pyridinium and quaternary ammonium	467	629	Plasma membrane	Cell membrane imaging	[66]
TTVP	Pyridinium and quaternary ammonium	480	708	Plasma membrane	Cell membrane imaging and PDT	[66]
TPBT	Pyridinium	440	537c) & 620	Green emission: dsDNA; red emission: dsDNA and other polyanionic analytes	Single‐nucleotide polymorphisms detection by dsDNA recognition	[68]
TPE–4EP+	Pyridinium	392	615	Mitochondria and nucleus	Self‐reporting PDT	[69]
TEPP	Pyridinium and triphenylphosphonium	503	672	Mitochondria	Mitochondrial imaging and PDT	[71]
PD–BZ–OH	Pyridinium and hydroxyl	410	596	Amyloid fibril	3D mapping of amyloid‐*β* plaques in mouse whole brain	[72]
PD–NA–OH	Pyridinium and hydroxyl	410	660	Amyloid fibril	Detection of amyloid‐*β* plaques in cells and tissue slides	[72]
TPEBAI	Sulfonate	350	625	Azide group	Bioorthogonal tumor labeling	[74]
QM–FN–SO_3_	Sulfonate	470	720	Amyloid fibril	In situ mapping of amyloid–*β* plaques	[79]
QM–SO_3_–ER	Sulfonate	450	540	K_ATP_ channel	Endoplasmic reticulum imaging	[80]
BCN–TPET–TEG	Triethyleneglycol (TEG)	460	700c)	Azide group	Bioorthogonal tumor labeling and image‐guided PDT	[84]
PEG–azo–PS4	PEG (≈2.0 kD)	418b), c)	642c)	Reductases in hypoxic tumors	Hypoxia‐mediated tumor imaging and PDT	[85]
AIE‐4PEG550	PEG (0.55 kD)	645	893	Kidney and bladder	Noninvasive diagnosis of kidney fibrosis	[42]
QM–HBT–*β*gal	Galactose	460b), c)	620c)	*β*‐gal	Long‐term tracking of endogenous *β*‐gal activity	[88]
TBPG	Glucose	440c), d)	580c)	*β*‐Glu	Soil enzyme analysis	[89]
GlcNAc–TPE	*N*‐acetyl‐*β*‐d‐glucosaminide	360b), c)	612c)	Hex	In situ visualization of Hex	[90]
mAb–CSPP	Cetuximab	405	600c)	EGFR	Bioimaging of specific cancer cells	[92]
TPE–R–DNA	Single‐strand DNA	428	620c)	MnSOD mRNA	Renal cancer imaging and prognosis analysis	[94]
AIE–Pep–DOX (APD)	DEVD and CREKA peptides	490c), d)	700c)	Tumor pH and caspase‐3	pH‐responsive chemotherapy and tumor‐detained photodynamic‐immunotherapy of triple‐negative breast cancer	[95]

a) Measured in aqueous solutions.

b) Measured in the aggregate state.

c) Measured upon the addition of analytes.

d) Measured in organic good solvents.

In summary, we have introduced the benefits of water‐soluble NIR AIEgens for highly accurate and sensitive phototheranostics. As the fields of chemistry, biology, and medicine continue to evolve and intersect, we anticipate the development of more advanced water‐soluble NIR AIEgens that will drive progress in fundamental biological research and translational medicine. We hope this perspective can offer our preliminary viewpoints to attract more researchers working in this field.

## Conflict of Interest

The authors declare no conflict of interest.
